# Analysis of Floral Organ Development and Sex Determination in *Schisandra chinensis* by Scanning Electron Microscopy and RNA-Sequencing

**DOI:** 10.3390/life12081260

**Published:** 2022-08-18

**Authors:** Xiuyan Liu, Lifan Zhang, Shihai Yang

**Affiliations:** 1 College of Chinese Medicine Materials, Jilin Agricultural University, Changchun 130118, China; 2 School of Life Sciences, Tonghua Normal University, Tonghua 134000, China

**Keywords:** *S. chinensis*, RNA-seq, sex determination, floral organ, scanning electron microscopy

## Abstract

*S. chinensis* is a typical monoecious plant, and the number and development of female flowers determines the yield of *S. chinensis*. Due to a lack of genetic information, the molecular mechanism of sex differentiation in *S. chinensis* remains unclear. In this study, the combination of scanning electron microscopy (SEM) and RNA sequencing (RNA-seq) was used to understand the way of sex differentiation of *S. chinensis* and to mine the related genes of sex determination. The result shows the development of male and female *S. chinensis* flowers was completed at the same time, the unisexual *S. chinensis* flowers did not undergo a transition stage between sexes, and sex may have been determined at an early stage in flower development. The results of the gene function analysis of the plant hormone signaling pathway and sucrose metabolism pathway suggest that auxin and JA could be the key hormones for sex differentiation in *S. chinensis*, and sucrose may promote pollen maturation at the later stage of male flower development. Two AGAMOUS (*GAG*) genes, 10 AGAMOUS-like MADS-box (AGLs) genes, and the MYB, NAC, WRKY, bHLH, and Trihelix transcription factor families may play important roles in sex determination in *S. chinensis*. Taken together, the present findings provide valuable genetic information on flower development and sex determination in *S. chinensis*.

## 1. Introduction

*Schisandra chinensis* (*S.* *chinensis*) is a woody single-leaf vine belonging to the Magnoliaceae family, and it is widely growing in northeast China, Korea, Japan, and other places in Asia. The fruit is bright red; the skin and pulp taste sour and sweet; and the kernel is spicy, bitter, and salty. Therefore, *S. chinensis* is called “wu wei zi” in China [1]. Modern pharmacological studies have shown that *S. chinensis* extract and *S. chinensis*-derived compounds have anti-cancer, antioxidant, neuroprotective, liver protective, and anti-inflammatory properties [2]. As *S. chinensis*-derived products, including fruit wine, fruit vinegar, and condiments are continually developed, *S. chinensis* has become a medicinal and edible plant with huge market prospects. As a typical monoecious plant, the number of *S. chinensis* male flowers is much higher than the number of female flowers; therefore, the yield of *S. chinensis* is not stable. For this reason, there is great interest in studying *S. chinensis* development and sex determination to improve yield. Previous studies on *S. chinensis* development have mainly focused on the chemical components and related biological activities, and no studies on sex determination in *S. chinensis* have been reported.

Flowers are important functional organs in plants that play a key role in reproduction and evolution. Flowers are generally divided into unisexual flowers and bisexual flowers. Among unisexual flowers, there are two types of sex differentiation. Type I unisexual flowers, such as Diospyros, are unisexual flowers that undergo pistil or stamen abortion to form unisexual flowers [3,4]. Type II unisexual flowers, such as *S. nigra*, do not go through a bisexual flower stage; rather, sex differentiation is completed by the beginning of flower bud development [5,6]. Scanning electron microscopy (SEM) can be used to finely observe the occurrence and development of flower organs during each round. This approach has been applied many times, including for *Jatropha* [7], *Adonis amurensis* [8], and *Juglans mandshurica* [9]. Therefore, SEM can both clearly visualize *S. chinensis* development and reveal whether *S. chinensis* undergoes Type I or Type II unisexual flower formation. 

Studying sex-determining genes in plants is technically difficult, and sex determination research has only progressed in a few plants. For example, ThdAG2, ThdAP3-2a, and ThdAP3-2b are presumed to be sex-determining genes in *Thalictrum dioicum* [10]. The class B genes *SpPISTILLATA* (*SpPI*) and *SpAPETALA3* (*SpAP3*) are considered male determinant genes in spinach [11]. In recent years, RNA-sequencing has emerged as an effective method to study flower development and sex determination, and the sex determination regions and genes in many plant species have been identified. For instance, 11 candidate genes for sex determination were screened in *Trichosanthes* [12], and in Persimmon, the regulatory network of the sex-determining gene MeGI was revealed [13]. In plants, such as *Asparagus officinalis* [14], *Vernicia fordii* [15], and spinach [16], a large number of genes related to sex determination have been identified through RNA-sequencing. Therefore, it is feasible to mine sex determination genes in *S. chinensis* using RNA-sequencing. It has also been shown that transcription factors, such as WRKY, MYB, and bHLH may play crucial roles in plant sex determination [17,18,19].

Plant hormones are a key factor in sex determination, and multiple hormones are known to be involved [20]. Among them, ethylene is the most effective hormone for sex regulation in cucurbitaceae plants. Sex-determining genes (CmWIP1, CmACS11, CmACS7) have been isolated, and a sex regulation pattern related to the ethylene synthesis pathway has been established [21]. Gibberellins (GA), jasmonic acid (JA), and Brassinolides (BRs) have male-promoting effects in a few unisexual flower plants [22], while CTKs can promote female flower development [23]. The present study observed the morphogenesis of flower organs in *S. chinensis* and mined for flower identity genes, transcription factors, and phytohormones related to sex determination. This work can provide a reference for future studies on the sex differentiation mechanism of *S. chinensis*.

## 2. Materials and Methods

### 2.1. Plant Materials

The experimental material is five-year-old healthy *S. chinensis*, grown in the Medicinal Botanical Garden of Jilin Agricultural University (Changchun, China). Sample collection was divided into two parts: (1). *S. chinensis* bud samples were collected every three days during the flower bud differentiation period (July 2017 to November 2017) and reproductive differentiation period (March to May 2018). The reproductive dormancy period was sampled every 20 days from November 2017 to March 2018. For each sampling, more than 30 buds were randomly collected and fixed in FAA (70% ethanol: acetic acid: formaldehyde, 90:5:5, *v*/*v*) to observe the morphological process of *S. chinensis* flower differentiation. Samples were collected from the first batch of buds developed in July. The largest bud in each cluster was selected for collection and tracking to ensure coherence in the collected buds’ developmental period, so that each stage of the development of *S. chinensis* could be observed. (2). Male and female flower buds of *S. chinensis* were collected on 8 May 2018 for RNA-seq. Twenty female flower buds were pooled as a biological replicate for each group, with a total of 3 biological replicates. The same process was carried out with male samples. When the flower bud was cut, the green pistil could be observed in the female bud, and the white stamen in the male flower bud. Collected samples were promptly frozen in liquid nitrogen and then stored in a −80 °C freezer.

### 2.2. Scanning Electron Microscopy Sample Processing

First, the samples were continuously dehydrated with 30, 50, 70, 85, 90%, 95% ethanol for 40 min in each step, and 100% ethanol for 50 min. Then, samples were soaked twice for 50 min with acetone, and then soaked twice for 30 min with isoamyl acetate. Next, the samples were subjected to CO_2_ critical point drying and then vacuum sprayed with gold. After sample preparation, the morphological development of flowers was observed under SEM (Hitachi S-3000N, Japan). All flower bud samples collected from late March to May 2018 were directly observed with a SZX10 stereo microscope. Judgment of the developmental stage of *S. chinensis*: the standard of more than 2/3 of the developmental morphology of each sample (30 bud samples) was the same.

### 2.3. RNA Extraction and Next Generation Sequencing

Total RNAs were extracted from six male and female flower samples using TRIzol reagent (Takara, Dalian, China). RNA quality was checked by an Agilent 2100 Bioanalyzer (Agilent Technology Company, Palo Alto, CA, USA) and NanoDrop2000 (Thermo Scientific, Wilmington, DE, USA). Equal amounts (3 μg) of RNA were extracted from each of three female flower samples, then pooled together to construct a paired-end sequencing library. The same process was carried out with three male samples. cDNA libraries were constructed from 2μg of mixed RNA using the NEBNext^®^ Ultra™ RNA Library Prep Kit for Illumina^®^ (NEB, Ipswich, MA, USA), and sequencing on the Illumina HiSeq 2000 platform: raw reads generated by Illumina instrument software were analyzed for the quality of initial sequence data using bcl2fastq16 (v2.17.1.14) and FastQC (v0.10.1) software to obtain filtered data [24]. Adapters and low-quality sequences of filtered reads were removed using Cutadapt17 (version 1.9.1) software to obtain clean data for subsequent de novo assembly (Trinity19 v2.2.0). Duplicate contigs were removed by cd-hit to obtain unigene sequence files [24].

### 2.4. Gene Function Annotation and Differential Gene Expression Analysis

Open reading frames were predicted using TransDecoder. All unigenes were annotated to the Nr, COG, GO, Swiss-Prot, and KEGG databases using the BLASTX program. The FPKM (fragments per kilobase per million reads) of the rsem (V1.2.4) software package was used to calculate the gene expression, with a high FPKM value, indicating high expression [25]. DESeq2 of the Bioconductor software package was used for differential gene expression (DEGs) analysis [26]. False discovery rates were controlled using Benjamini and Hochberg. Differential genetic screening was performed with |log2FoldChange| > 2 and FDR ≤ 0.05. GO and KEGG functional enrichment of DEGs by Goatools software and R script. The corrected *p* value was used as the threshold, and when the *p* value was <0.05, the GO function and the KEGG pathway function were considered to be significantly enriched.

### 2.5. Validation of RNA-seq by RT-qPCR

Validation of RNA-Seq results by RT-qPCR analysis of 9 DEGs (STIGAM, CRABS, GAG, TS2, NCED3, FL-54, YABBY, A6, and STAMEN). To verify the reliability of the RNA-seq results, samples from the same period as RNA sequencing were selected as experimental materials. The qRT-PCR assays were performed using a qTOWER^3^ thermal cycler (Analytik Jena AG, Jena, Germany) and SYBR Premix ExTaq II kit (Takara, Kyoto, Japan). The RT-qPCR reaction was conducted at 95 °C for 30 s, 40 cycles of 95 °C for 5 s, 60 °C for 35 s and 72 °C for 15 s. Data were analyzed using the 2^−∆∆CT^ method [27]. The RNA extraction and cDNA synthesis used in this part of the experiment were the same as used in 2.3. Gene-specific primers were designed by Primer Premier 5 software using TUBA as the reference gene. The primer sequences are shown in Table S11, which were synthesized by BGI (http://www.bgi-write.com/page/index/#/t/home/) (accessed on 1 August 2020), Using three biological and technical replicates.

## 3. Results

**Figure 1:**
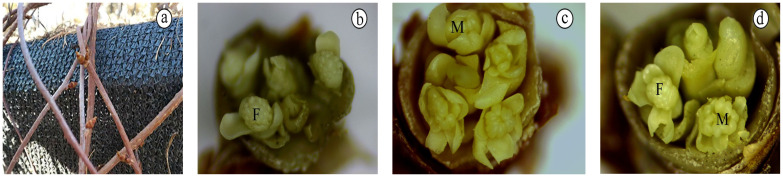
(**a**) Flower buds; (**b**) All female flower buds; (**c**) All male flower buds; (**d**) Mixed male and female buds. Abbreviation: F: female flower; M: male flower.

The characteristics of *S. chinensis* flower development: Mid-July 2017 to April 2018, flower development was carried out within the bud (Figure 1a). When the scales on the bud surface were removed, three floret types could be observed within the bud. That is, some buds had both female and male flowers, some buds contained entirely female flowers, and some buds contained entirely male flowers (Figure 1b–d). The number of florets was typically four to five, although a few buds had six to eight flowers. In middle-to-late April of the following year, the buds swelled and opened, the scales loosened and cracked, and flower buds appeared. Subsequently, the flowers continued to develop until they entered the flowering stage in mid-May.

### 3.1. Development of Floral Organ

**Figure 2:**
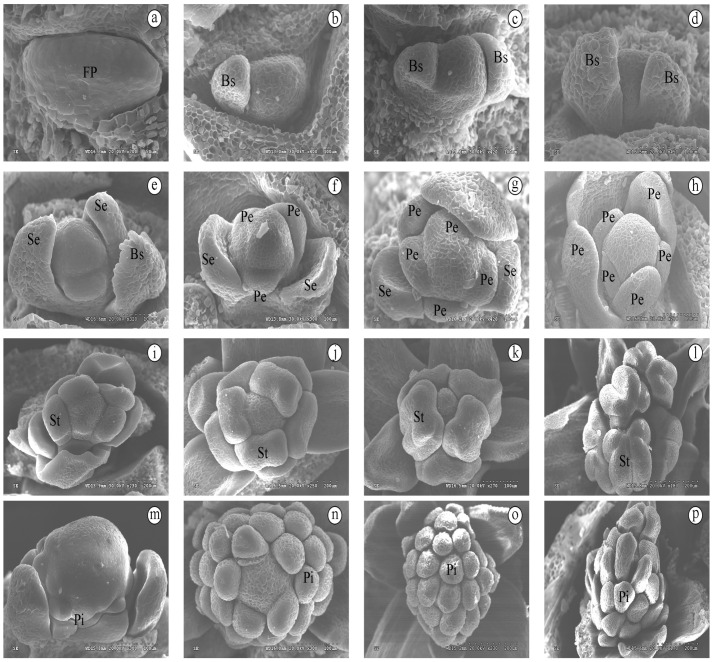
Developmental morphology of *S. chinensis* from July to November. (**a**). Flower primordium; (**b**–**d**). Bud scales development; (**e**,**f**). The development of sepals and hairy structure on the top of bud scales; (**g**,**h**) Two rounds of petal development; (**i**). Differentiation of stamen primordium; (**j**,**k**) Appearance of tetrahedral rudimentary anthers; (**l**) Anther chamber, fully developed stamens of anther septum. (**m**) Initiation of the pistil primordia; (**n**,**o**) Carpel primordia arranged spirally; (**p**) Carpel elongation, plicate carpels. Abbreviation: FP. floral primordia; Bs. bud scale; Se. sepal; Pe. petal; Pi. pistil; St. Stamen. Scale. (**a**). 50 μm**,** (**b**–**i**,**k**,**n**) 100 μm, (**j**,**l**,**m**,**o**,**p**). 200 μm.

In mid-July, flower primordia appeared (Figure 2a). Subsequently, two bud scales of different sizes differentiated on both sides of the flower primordium (Figure 2b–d). In late July, the flower primordia continued to develop, the top became taller and wider, the surface became round and blunt, and the volume became larger. The two bud scales continued to grow, and hairy structures appeared at the top of the bud scales (Figure 2e,f). Meanwhile, two sepals developed at the base of the scales. Afterwards, on the inside of the sepals, the first and second rounds of petals developed (Figure 2g,h). No signs of sexual organ development in female and male flowers were observed during this period. In mid-August, stamen and pistil differentiation in *S. chinensis* proceeded simultaneously. In male flowers, the male primordia continued to expand, and the five stamen primordia gradually differentiated to form stamens with four pollen sacs (Figure 2i–l). In female flowers, the pistil primordium proliferated and became large and nearly spherical, and the carpel primordium was closely arranged (Figure 2m–p). Each carpel was plicately formed (Figure 2p).

### 3.2. Continuing Development of Floral Organ

**Figure 3:**
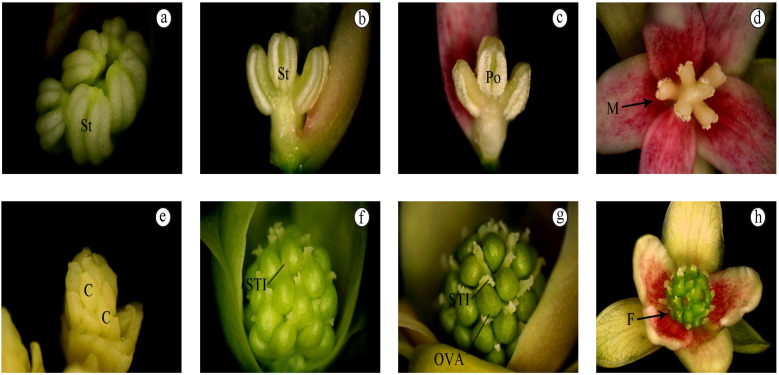
Continued development of female and male flowers. (**a**–**d**) Apical and lateral view of stamen development; pollen maturation and development; (**e**–**h**) The development of pistil and stigma. Abbreviation: St. stamen; Po. pollen; C. carpel; STI. stigma; OVA. ovary; F. female; M. male.

In mid-April of 2018, the flower buds of *S. chinensis* entered the secondary development stage. The stamens eventually developed into Monadelphous; that is, five stamens separated independently, and the filaments were columnar (Figure 3a,b). In early May, the pollen aperture cracked and released mature pollen (Figure 3c,d). The female flower carpel further developed to form a subspherical young ovary (Figure 3e,f), and the stigma extended and became longer, developing into a bifurcated, semi-curled morphology. Soon after, anthesis occurred (Figure 3g,h).

### 3.3. Overview of RNA-Seq Sequencing

**Figure 4:**
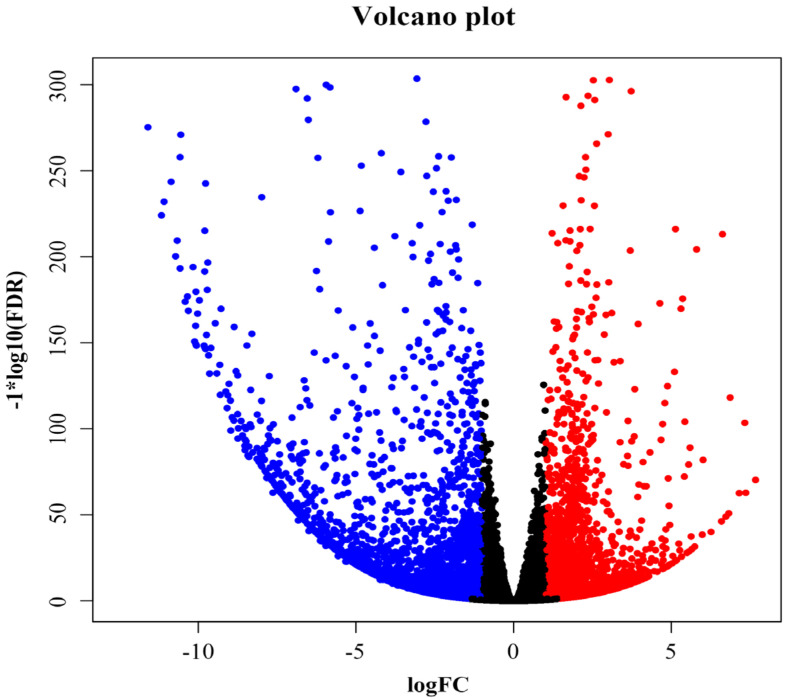
Volcano plot of differentially expressed genes. Blue: down, Red: up. Abscissa: fold change in gene expression; Vertical axis: Statistical significance of differences in gene expression changes.

To identify the molecular regulation of sex differentiation in *S. chinensis,* the female and male flower buds of *S. chinensis* were sequenced. A total of 62.7GB of data was obtained, and the filtered data are shown in Table S1. The average Q30 value of two samples was higher than 95%, and the average Q20 value of the same two samples was higher than 98%, indicating that the quality of the filtered data reads was good and could be used for subsequent analyses. After de novo assembly, 16,180,003 contigs were obtained with an average length of 46.50 bp and an N50 length of 47 bp. After sequence assembly and de-redundancy treatment, 256,452 unigenes were obtained, with an average length of 579.20 bp, an N50 length of 805 bp, and a G+C content of 40.85% (Table S2). Concerning unigene length distribution, 200–500 bp accounted for the largest proportion, 69.74%, and greater than 2000 bp accounted for 4.38% (Table S3). DEGs from female and male flowers were screened for more than 2-fold changes in differential gene expression and false discovery rate (FDR) ≤ 0.05. Substantial transcriptional differences were observed in pairwise comparisons between female and male flowers. We used the *S. chinensis* unigene of male flowers as the control compared to unigene of females, and a comparison of the data for the male flowers and female showed that 10,977, 4392 (39.94%), and 6605 (60.06%) genes were differentially expressed, up-regulated, and down-regulated, respectively (Figure 4 and Table S4).

### 3.4. Differential Genes Function Analysis

**Figure 5:**
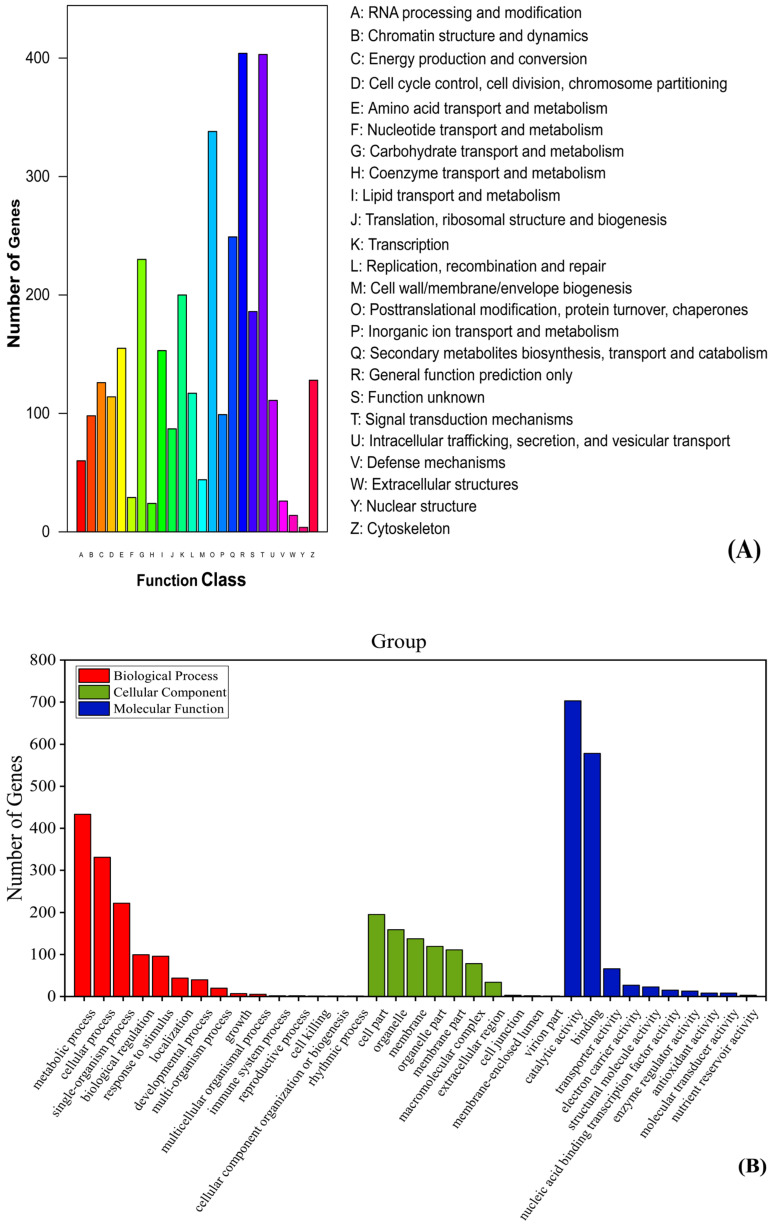

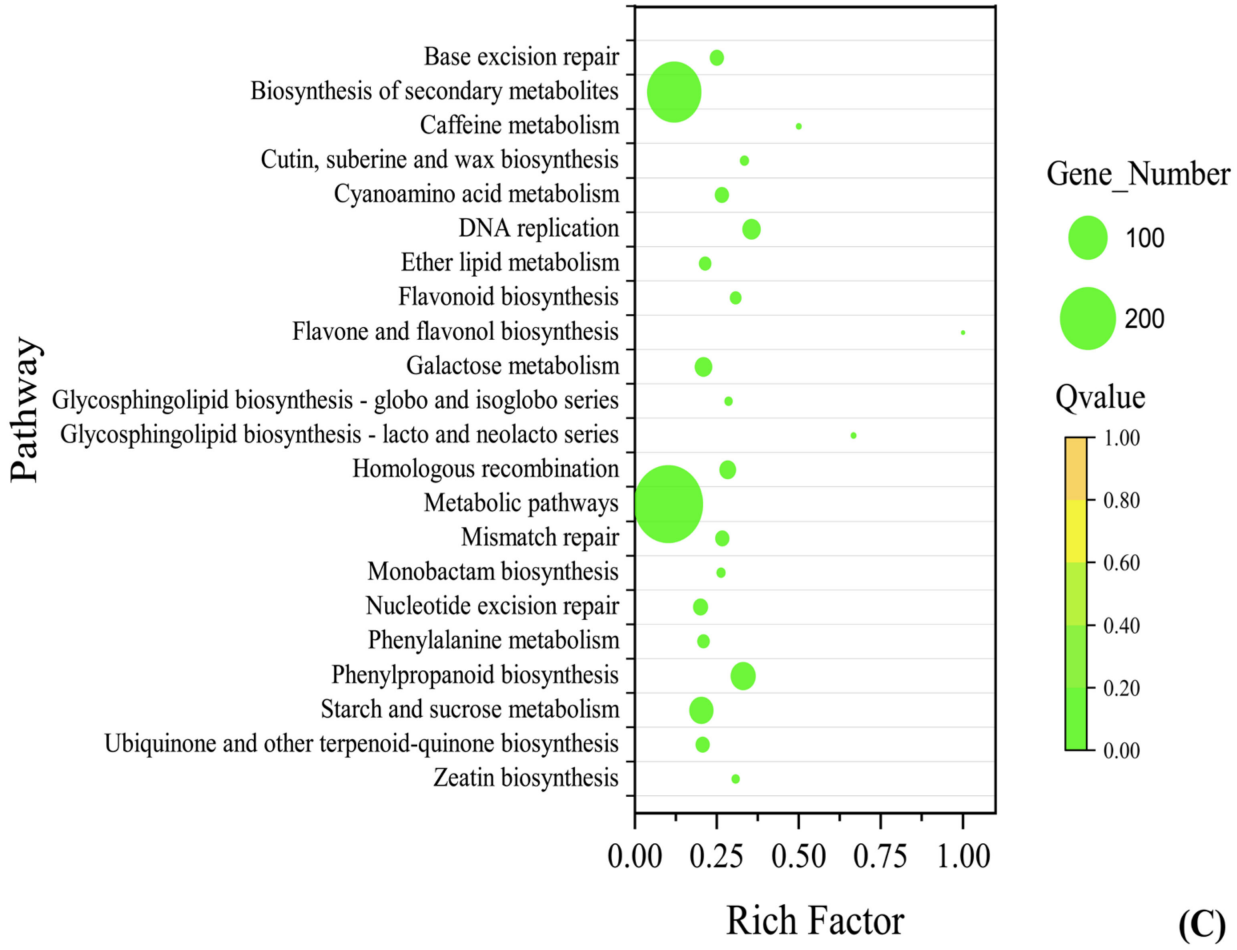
Differential gene functional analysis. (**A**) COG Function classification map. The y-axis shows the number of gene and the x-axis the class; (**B**) Histogram of GO enrichment. Three colors represent three categories; (**C**) The KEGG enrichment scatter plot. The larger the Rich factor value, the greater the degree of enrichment. 0 < Q < 0.05, the closer the Q value is to green, the more significant the enrichment.

All DEGs were matched to the COG database to further validate protein function and classification. A total of 3399 DEGs were mapped to 25 functional categories in the COG database, and this covered most life processes (Figure 5A and Table S5). The most enriched gene categories were “General function prediction only” (404, 11.89%), “Signal transduction mechanisms” (403, 11.86%), and “Posttranslational modification, protein turnover, chaperones” (338, 9.9%). Secondly, “Secondary metabolites biosynthesis, transport and catabolism” (249, 7.3%), “Carbohydrate transport and metabolism” (230, 6.8%), “Transcription” (200, 5.89%), and “Amino acid transport and metabolism” (155, 4.6%). Finally, “Lipid transport and metabolism” (153, 4.5%), “Cytoskeleton” (128, 3.7%), “Energy production and conversion” (126, 3.7%) and “Cell cycle control, cell division, chromosome partitioning” (114, 3.4%).

The GO database includes three categories in total: biological process (BP), cellular component (CC), and molecular function (MF). These three functions describe the possible molecular functions of gene products, the cellular environment in which they are located, and the biological processes in which they are involved. A total of 3587 DEGs were annotated into 35 subcategories in the GO database (Figure 5B and Table S6). In BP, the top three terms were “metabolic process” (GO:0008152), “cellular process” (GO:0009987) and “single-organism process” (GO:0044699), with 433, 331, and 222 unigenes, respectively. In MF, “binding”(GO:0005488), “catalytic activity” (GO:0003824), and “transporter activity” (GO:0005215) had the highest abundance, with 578, 703, and 66 unigenes, respectively. In CC, genes were mainly concentrated in three subclasses, including “cell part” (GO:0044464), “organelle” (GO:0043226), and “membrane” (GO:0016020), with 195, 159, and 137 unigenes, respectively.

All DEGs were aligned to the KEGG database to further explore the metabolic pathways through which the DEGs regulate flower development. The annotation results showed that a total of 627 differential genes were significantly enriched in 22 pathways in the two categories of metabolism (551, 87.88%) and genetic information processing (76, 12.12%) (Figure 5C and Table S7). The pathways with the most annotated genes were the “ko01100” metabolic pathway, (294, 46.89%) and “ko01110” secondary metabolite biosynthesis (178, 28.39%), followed by “ko00940” phenylpropane biosynthesis (39, 6.22%) and “ko00500” starch and sucrose metabolism (36, 5.74%). These annotations provide a data basis for further research in exploring genes associated with *S. chinensis* female and male flower development.

### 3.5. Identification of Genes Associated with Flower Development from DEGs

The formation and sex differentiation of floral organs are accomplished by the precise coordination of many genes. Candidate genes were screened according to the annotation results from public databases (NR, COG, GO, Uniprot, KEGG). MADS-box genes play important regulatory roles in floral organogenesis, differentiation, and morphogenesis. A total of 16 MADS-box family members were differential expression in female and male flowers, including two *AGAMOUS* (*GAG*) (GO:0006355) genes, ten AGAMOUS-like (AGLs) genes, two *SOC1* genes, one *APETALA3-2* gene, and one *GGM13* gene. The expression of these genes was significantly different between female and male flowers, suggesting that these genes may play an important role in sex differentiation in *S. chinensis*. In addition, three carpel development genes (*CRABS*) and two stigma-specific genes (*STIG1-like*) were highly expressed in female flowers. Interestingly, we identified three differentially expressed *TS2* genes, which are key genes in maize sex determination. Whether these *TS2* genes regulate sex determination in *S. chinensis* requires further study. Many genes involved in floral developmental pathways were also screened, including *EFM*, *ERF*, the AP2-like ethylene-responsive transcription factors (*ANT*, and *AIL*), *ULTRAPETALA*, *WUSCHEL*, *TT12*, *ASHR3*, and *KANADI* (Table S8).

Although no hormone synthesis pathway genes were significantly enriched in the KEGG map, according to the gene function annotation, a total of 31 genes were enriched in the plant hormone signal transduction pathway “ko04075”. These genes were primarily JA and auxin synthesis pathway genes, including jasmonic acid-amino synthetase (*JAR1_4_6*), auxin influx carrier (*AUX1*, *LAX*)*,* auxin responsive GH3 gene family (*CH3*), SAUR family protein (*SAUR*)*,* auxin response factor (*ARF*)*,* and auxin-responsive protein IAA (*IAA*). Notably, the 11 highly expressed genes in female flowers were related to auxin, whereas the genes related to JA were highly expressed in male flowers (Table S9). Sucrose is a carbohydrate and a signaling molecule that regulates plant growth and development [28]. Thirty-six genes in the starch and sucrose metabolism “ko00500”pathway were significantly enriched in KEGG (Figure 5 and Table S9). Including 12 beta-glucosidase genes; there were 5 beta-amylase and beta-fructofuranosidase each; there were 3 trehalose 6-phosphate phosphatase, star synthase, and glucose-1-phosphate adenylyltransferase each; and there was 1 each of endoglucanase, sucrose-phosphate synthase, fructokinase, and hexokinase. In addition, 27 carbon metabolism “ko01200” pathway genes were enriched in the differential gene KEGG database. Among these sugar-related genes, only three enzyme genes were up-regulated in female flowers, and the others were highly expressed in male flowers.

**Figure 6:**
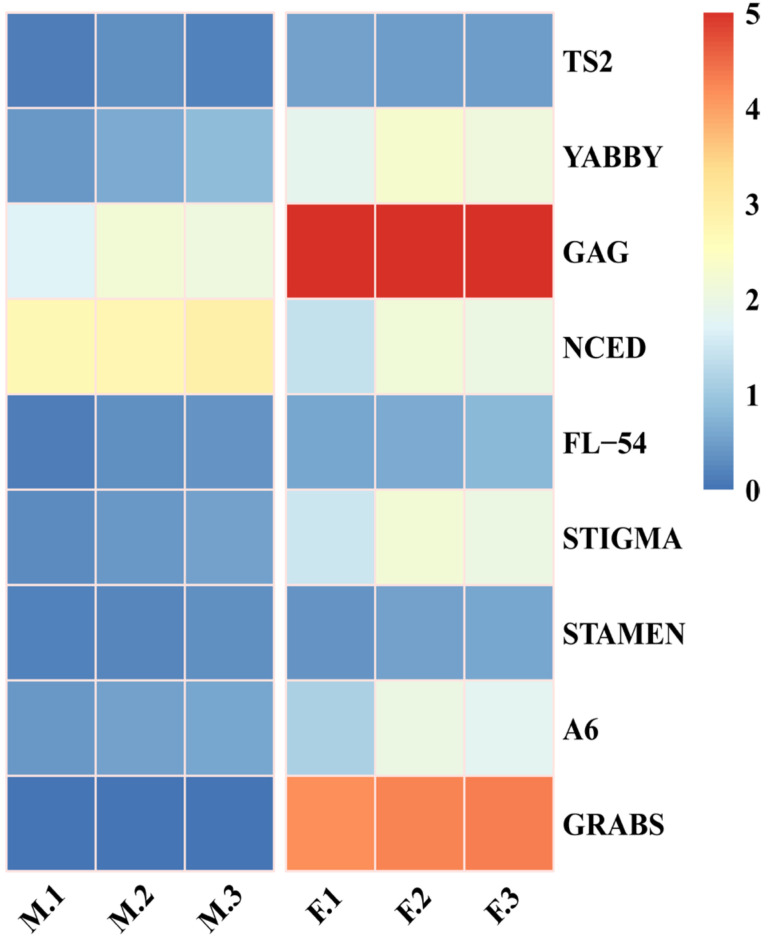
RT-qPCR gene expression profiles of nine genes. F, M: female and male flower samples, 1, 2, 3: three biological and technical replicates. The blue to red color scale indicates gene expression levels from low to high.

TFs are involved in flower development and sex determination by regulating genes in different pathways [18]. Two-hundred-fifty-nine transcription factors related to flower development were identified in DEGs. These genes belonged to 17 transcription factor families (Table S10). Among them, the top four transcription factor families were MYB (59), bHLH (42), NAC (37), and WRKY (21). These findings will help to mine the sex determination-related genes of *S. chinensis*. Furthermore, nine genes were selected from the DEGs for RT-qPCR validation (Figure 6). The results showed concordance between RNA-Seq and RT-qPCR expression analysis, which confirmed the reliability of the transcriptomic data.

## 4. Discussion

The process of flower formation in *S. chinensis* took 10 months. Long flower development processes have been observed in other species, including *Vernicia fordii* [15], *Ixora chinensis* [29], apple [30]. However, the formation of each plant floral organ has its unique differentiation pattern. SEM showed that the development of female and male flowers in *S. chinensis* was synchronized. Throughout flower development, female organs (carpels) were never observed in male flowers, and male organs (anthers) were never observed in female flowers. This indicates that the unisexual *S. chinensis* flowers did not undergo a transition stage between sexes, and sex may have been determined at an early stage in flower development.

To further reveal the key sex differentiation genes in *S. chinensis*, RNA-seq was performed on male and female flower buds, and DEG function was analyzed to mine genes related to sex determination. The formation of sepals, petals, pistils, and stamens can be well explained according to the ABCDE model of floral organ development. Among them, B and C genes not only determine the development of pistil and stamen but are also key genes in sex differentiation [16]. In the present study, two class C genes AGAMOUS (*GAG*) were highly expressed in female flowers. Meanwhile, three genes *CRABS CLAW* (*CRC*) were only expressed in female flowers. *LEAFY* (*LFY)*, *WUSCHEL* *(WUS)*, and *KANADI* *(KAN)* were also upregulated in female flowers. It has been previously shown that the *CRC* gene is required to control carpel development in *Arabidopsis thaliana* [31]. This gene not only inhibits the early radial growth of pistils and promotes its later elongation, but it also participates in the entire growth and maturation process of nectary [32]. Although *CRC* can independently direct carpel development, *AG* can enhance its expression [33]. The gene *LFY* is a direct factor for *AG* activation [34], and *WUS* is a key gene for the termination of floral meristems [35]. The cooperation of *WUS* and *LEY* can directly activate the expression of *AG*, and the overexpression of *WUS* can reduce the demand for *LFY* during AG activation [36,37]. WUS induces the expression of *AG*, and the expression of *AG* generates a negative feedback loop to inhibit the expression of *WUS*, which causes the cells in the center of the floral tissue differentiate into carpels [38]. The SAND domain protein *ULTRAPETALA1* not only prevents excessive floral organ and carpel production but also activates *AG* expression with the *LFY* gene [39,40,41]. KAN is a regulator of organ polarity in *Arabidopsis* [42] that interacts with *ULT1* to restrict the activity of the pistil apical promoter and promote the development of pistil tissue [43]. *AG* is also critical for anther differentiation during the later stages of flower development [44]. In addition to the *AG* genes, 10 *AGL* genes were also identified in this study. Three of these genes were highly expressed in male flowers, and the other seven *AGLs* were highly expressed in female flowers. *AGLs* are class C genes of the *AG* subfamily that promote flowering and regulate pistil and stamen development in flowering plants [45,46,47]. These results suggest that *GAG* and *AGLs* may promote female flower development by positively regulating the expression of these genes.

TFs are essential for plant growth and organ development. RNA-seq analysis revealed that multiple TF families were significantly differentially expressed in female and male flowers of *S. chinensis*. According to the known functions of transcription factors in flower development, five transcription factor families, MYB, NAC, bHLH, WRKY, and Trihelix, attracted our attention. MYB is one of the largest transcription factor families in plants. In the process of flower organ development, MYB family genes affect the development of stamens by regulating tapetum development, pollen development, and anther dehiscence [48,49,50,51]. For example, the R2R3-MYB transcriptional activator BcMF28 encoded in rapeseed is specifically expressed in *Arabidopsis* stamens. Its overexpression causes defects in stamen development, including shortened filaments, non-dehiscence of anthers, and pollen abortion, ultimately leading to male sterility [49]. Interestingly, several reports indicate that NAC family genes can interact with MYB family genes to regulate anther development during the development of floral organs [52,53,54]. The bHLH family is also an important transcription factor family involved in the morphogenesis of plant flowers. The bHLH transcription factor SPT promotes the growth of carpel margins and pollen shells [55]. Loss of function mutations in *SPT* result in severely impaired ovary and style development and reduced stigma tissue [56]. The bHLH factors encoded by the *HEC* genes form dimers with SPT and together regulate the development of female reproductive tissues [57]. The *DYT1* and *AMS* genes encode bHLH transcription factors. In the genetic pathway of tapetum development, *DYT1-TDF1-AMS- MS188-MS1*, *DYT1* directly regulates the expression of *TDF1*, thereby affecting tapetum development and pollen wall formation [58,59]. WRKY transcription factors can also promote plant flowering [60]. It has been shown that *MlWRKY12* can upregulate *CO, FT, LFY*, and other flowering-related genes to regulate the flowering time of *Miscanthus lutarioriparius* [61]. Other members of the WRKY family, such as *WRKY13*, *WRKY71*, *WRKY75*, can also promote plant flowering through the age pathway and the gibberellin pathway [62,63,64]. Notably, the Trihelix family gene *SlGT11* is specifically expressed in the primordia corresponding to the tomato stamens and carpels. It has been shown that *SIGT11* can induce reversal from reproductive to vegetative flower development, providing further evidence for the importance of *SlGT11* in flower organ development [65]. In the present study, seven Trihelix family genes were differentially expressed in female and male flowers, whether these genes play an important role in *S. chinensis* sex differentiation requires further study. Our findings suggest that these TF families transcription factors may be involved in *S. chinensis* sex differentiation.

Plant hormones are the most important endogenous signaling molecules in floral organ development and sex differentiation. Examples of important plant hormones include JA in corn [66], ethylene in cucumber [21], and BR in spinach [18]. In the present study, the highly expressed genes in female flowers were all associated with auxin synthesis (11 genes), and most of the highly expressed genes in male flowers were associated with auxin (5 genes) and JA synthesis (3 genes). Among the highly expressed genes in male flowers, four were CH3 genes belonging to the auxin responsive GH3 gene family. In a previous study, the knockout of the *OsMGH3* gene in rice anthers altered stamen development and pollen viability and resulted in decreased fertility. High *OsMGH3* expression in rice florets contributes to carpel and anther development and affects floret fertility by regulating auxin activity [67]. AG also coordinates late stamen maturation by controlling expression of genes involved in plant hormone JA biosynthesis [44]. Many prior studies have also reported the involvement of *MYB* family genes in anther development via the auxin and JA pathways [68,69,70,71,72,73]. Based on their roles in flower development in other species, we can infer that auxin and JA may contribute to male flower development in *S. chinensis*.

Carbohydrates are a vital component of flowering regulation. In this study, KEGG significantly enriched the “starch and sucrose metabolism” (ko00500) pathway, with a total of 36 enzyme genes of 11 types (Figure 5 and Table S9). Several of these enzyme genes have been studied, and it has been confirmed that they affect flowering. For example, three genes involved in sucrose metabolism (SuSy, UGPase and SPS) were introduced into tobacco plants, and it was found that these genes not only directly affected the internode growth of tobacco, but also delayed the flowering time of tobacco [74]. The fructokinase FRK3 and FRK1 genes of *Arabidopsis* are delayed in flowering during short-day conditions [75]. TPS1 (trehalose 6-phosphate phosphatase) was also found to act as a flowering-inducing signal gene that affects timely flowering in Arabidopsis by promoting the flowering signal downstream of the flowering locus T (FT) [76]. In addition to this, studies have shown that fructokinase and hexokinase also have an effect on floral organ development. For instance, hexokinase (Os HXK10) in rice is especially expressed in stamens and plays an important role in anther dehiscence, pollen germination, and grain filling in rice [77]. By analyzing the tomato fructokinase gene (Sl FRK4) and other sugar metabolism genes, it was found that Sl FRK4 and the invertase LIN7 were co-expressed during pollen germination and maturation [78]. Furthermore, sucrose is required for both *Arabidopsis* pollen germination [79] and tobacco pollen tube growth [80]. The above analysis results indicated that sucrose may promote the flowering and pollen development of *S. chinensis* during the reproductive process of *S. chinensis.*

## 5. Conclusions

To summarize, *S. chinensis* flower development is a lengthy process. The development of male and female flowers is synchronized, and a unisexual flower forms without the process of abortion. The analysis of developmental genes in *S. chinensis* showed that the MODS-box family gene *GAG* may promote the development of female flowers by positively regulating the *CRC*, *LFY*, *WUSCHEL*, and *KANADI* genes. In addition, the *AGL* genes may also play important roles in *S. chinensis* sex differentiation. The MYB, NAC, and bHLH transcription factor families may be involved in anther development. The results of the gene function analysis of the plant hormone signaling pathway and sucrose metabolism pathway suggest that auxin and JA could be the key hormones for sex differentiation in *S. chinensis*, and sucrose may promote pollen maturation at the later stage of male flower development. Our findings provide a valuable resource for future studies of *S. chinensis* flower development and sex differentiation.

## Data Availability

All fastq raw sequence read data have been uploaded to the NCBI Sequence Read Archive (SRA) under accession number PRJNA599606.

## Supplementary Materials

The following supporting information can be downloaded at: https://www.mdpi.com/article/10.3390/life12081260/s1, Table S1: Data quality statistics after filtering; Table S2: Unigene quantity and length distribution statistics; Table S3: Unigene distribution ratio and quantity of different lengths; Table S4: List of differentially expressed genes; Table S5: COG enrichment analysis of the DEGs; Table S6: GO enrichment analysis of the DEGs; Table S7: KEGG enrichment analysis of the DEGs; Table S8: DEGs associated with flower development. Table S9: Genes related to plant hormones and glucose metabolism; Table S10: Different transcription factor families from the DEGs; Table S11: the primer sequence information for RT-qPCR.

## References

[1] Hancke J.L., Burgos R.A., Ahumada F. (1999). *S. chinensis* (Turcz.) Baill. Fitoterapia.

[2] Yang S.Y., Yuan C.H. (2021). *S. chinensis*: A comprehensive review on its phytochemicals and biological activities. Arab. J. Chem..

[3] Mayer S.S., Charlesworth D. (1991). Cryptic dioecy in flowering plants. Trends Ecol. Evol..

[4] Akagi T., Kajita K., Kibe T., Morimura H., Tsujimoto T., Nishiyama S., Kawai T., Yamane H., Tao R. (2013). Development of molecular markers associated with sexuality in *Diospyros lotus* L. and their application in D. kaki Thunb. J. Jpn. Soc. Hortic. Sci..

[5] Mitchell C.H., Diggle P.K. (2005). The evolution of unisexual flowers: Morphological and functional convergence results from diverse developmental transitions. Am. J. Bot..

[6] Sanderson B.J., Feng G.Q., Hu N., Carlson C.H., Smart L.B., Keefover-Ring K., Yin T.M., Ma T., Liu J.Q., DiFazio S.P. (2020). Sex determination through X-Y heterogamety in *Salix nigra*. bioRxiv.

[7] Anupharb S., Lucsame G., Tittinat P., Prapassorn D.E., Jantrararuk T., Siam P. (2018). Transcriptome analysis of *Jatropha curcas* L. flower buds responded to the paclobutrazol treatment. Plant Physiol. Biochem..

[8] Liu X.L., Li J.H., Zhu J.Y., Yang Y.F. (2016). Floral differentiation and growth rhythm of rhizome buds of the spring ephemeroid plant *Adonis amurensis* Regel et Radde. Phyton-Int. J. Exp. Bot..

[9] Zhang L.J., Guo C., Lu X.J., Sun X.M., Liu C.P., Zhou Q., Deng J.F. (2021). Flower Development of Heterodichogamous *Juglans mandshurica* (Juglandaceae). Front. Plant Sci..

[10] Di S.V., Kramer E.M., Baum D.A. (2005). Floral MADS box genes and homeotic gender dimorphism in *Thalictrum dioicum* (Ranunculaceae)—A new model for the study of dioecy. Plant J..

[11] Maja J., Noah S.D., Golenberg E.M. (2010). Functional analysis of B and C class floral organ genes in spinach demonstrates their role in sexual dimorphism. BMC Plant Biol..

[12] Hu X.Q., Liao Z.Y., Zhang B., Yue J.J., Wang Z., Jie X., Liu J. (2020). Transcriptome sequencing and screening of genes related to sex determination of *Trichosanthes kirilowii* Maxim. PLoS ONE.

[13] Yang H.W., Akagi T., Kawakatsu T., Tao R. (2019). Gene networks orchestrated by MeGI: A single-factor mechanism underlying sex determination in persimmon. Plant J. Cell Mol. Biol..

[14] Li S.F., Zhang G.J., Zhang X.J., Yuan J.H., Deng C.L., Gao W.J. (2017). Comparative transcriptome analysis reveals differentially expressed genes associated with sex expression in garden asparagus (*Asparagus officinalis*). BMC Plant Biol..

[15] Mao Y.J., Liu W.B., Chen X., Xu Y., Lu W.L., Hou J.Y., Ni J., Wang Y.T., Wu L.F. (2017). Flower Development and Sex Determination between Male and Female Flowers in *Vernicia fordii*. Front. Plant Sci..

[16] Li N., Wang Y.Y., Wang J.W., Zhang W.Q., Meng Z.W., Wang Y.S., Zhang Y.L., Li S.F., Gao W.J., Deng C.L. (2022). Identification of Sex Differentiation-Related microRNAs in Female and Male Flower. Int. J. Mol. Sci..

[17] Murase K., Shigenobu S., Fujii S., Ueda K., Murata T., Sakamoto A., Wada Y., Yamaguchi K., Osakabe Y., Osakabe K. (2017). MYB transcription factor gene involved in sex determination in *Asparagus officinalis*. Genes Cells Devoted Mol. Cell. Mech..

[18] Liu Z.Y., Wang H.Y., Xu Z.S., Zhang H.L., Li G.L., Wang X.W., Qian W. (2021). Transcriptome profiling of differentially expressed genes of male and female inflorescences in spinach (*Spinacia oleracea L.*). Genome.

[19] Li X., Han R., Cai K.W., Guo R.X., Pei X.N., Zhao X.Y. (2022). Characterization of Phytohormones and Transcriptomic Profiling of the Female and Male Inflorescence Development in Manchurian Walnut (*Juglans mandshurica* Maxim.). Int. J. Mol. Sci..

[20] Monika H., Kuldeep S., Manoj P., Veena A. (2015). Review on different mechanisms of sex determination and sex-linked molecular markers in dioecious crops: A current update. Euphytica.

[21] Boualem A., Troadec C., Camps C., Lemhemdi A., Morin H., Sari M.-A., Fraenkel-Zagouri R., Kovalski I., Dogimont C., Perl-Treves R. (2015). A cucurbit androecy gene reveals how unisexual flowers develop and dioecy emerges. Science.

[22] Zhang J.S., Adnane B., Abdelhafid B., Ray M. (2014). Genomics of sex determination. Curr. Opin. Plant Biol..

[23] Luo Y., Pan B.Z., Li L., Yang C.X., Xu Z.F. (2020). Developmental basis for flower sex determination and effects of cytokinin on sex determination in *Plukenetia volubilis* (Euphorbiaceae). Plant Reprod..

[24] Wang X.H., Hui F., Yang Y.C., Yang S.H. (2018). Deep sequencing and transcriptome analysis to identify genes related to biosynthesis of aristolochic acid in *Asarum heterotropoides*. Sci. Rep..

[25] Dewey C.N., Li B. (2011). RSEM: Accurate transcript quantification from RNA-Seq data with or without a reference genome. BMC Bioinform..

[26] Robinson M.D., McCarthy D.J., Smyth G.K. (2010). edgeR: A Bioconductor package for differential expression analysis of digital gene expression data. Bioinformatics.

[27] Pfaf M.W. (2001). A new mathematical model for relative quantification real-time PCR. Nucleic Acids Res..

[28] Braun D.M., Wang L., Ruan Y.L. (2014). Understanding and manipulating sucrose phloem loading, unloading, metabolism, and signalling to enhance crop yield and food security. J. Exp. Bot..

[29] Chen L.Y., Chu C.Y., Huang M.C. (2003). Inflorescence and flower development in Chinese ixora. Am. Soc. Hort. Sci..

[30] Foster T., Johnston R., Seleznyova A. (2003). A morphological and quantitative characterization of early floral development in apple (*Malus x domestica Borkh*). Ann. Bot..

[31] Gross T., Broholm S., Becker A. (2018). CRABS CLAW acts as a bifunctional transcription factor in flower development. Front. Plant Sci..

[32] Bowman J.L., Smyth D.R. (1999). CRABS CLAW, a gene that regulates carpel and nectary development in *Arabidopsis*, encodes a novel protein with zinc finger and helix-loop-helix domains. Development.

[33] Gómez-Mena C., Folter S., Rosta M.M. (2004). Transcriptional program controlled by the floral homeotic gene AGAMOUS during early organogenesis. Development.

[34] Michael L., Andrea B., Gerd J., Thomas L. (2001). Termination of Stem Cell Maintenance in *Arabidopsis* Floral Meristems by Interactions between WUSCHEL and AGAMOUS. Cell.

[35] Guo L., Cao X.W., Liu Y.H., Li J., Li Y.P., Li D.M., Zhang K., Gao C.X., Dong A.W., Liu X.W. (2018). A chromatin loop represses WUSCHEL expression in *Arabidopsis*. Plant J..

[36] Sablowski R. (2007). Flowering and determinacy in *Arabidopsis*. J. Exp. Bot..

[37] Lohmann J.U., Ray L.H., Martin H., Maximilian A.B., François P., Rüdiger S., Detlef W. (2001). A Molecular Link between Stem Cell Regulation and Floral Patterning in *Arabidopsis*. Cell.

[38] Cao X.W., He Z.S., Guo L., Liu X.G. (2015). Epigenetic Mechanisms Are Critical for the Regulation of WUSCHEL Expression in Floral Meristems. Plant Physiol..

[39] Cristel C.C., Jennifer C.F. (2009). The SAND domain protein ULTRAPETALA1 acts as a trithorax group factor to regulate cell fate in plants. Cold Spring Harb. Lab. Press..

[40] Carles C.C., Choffnes I.D., Reville K. (2005). ULTRAPETALA1 encodes a SAND domain putative transcriptional regulator that controls shoot and floral meristem activity in *Arabidopsis*. Development.

[41] Engelhorn J., Moreau F., Fletcher J.C., Carles C.C. (2014). ULTRAPETALA1 and LEAFY pathways function independently in specifying identity and determinacy at the *Arabidopsis* floral meristem. Ann. Bot..

[42] Kerstetter R.A., Bollman K., Taylor R. (2001). KANADI regulates organ polarity in *Arabidopsis*. Nat. Int. Wkly. J. Sci..

[43] Pires H.R., Shemyakina E.A., Fletcher J.C. (2015). The ULTRAPETALA1 trxG factor contributes to patterning the *Arabidopsis* adaxial-abaxial leaf polarity axis. Plant Signal. Behav..

[44] Ito T., Ng K.H., Lim T.S., Yu H., Meyerowitz E.M. (2007). The homeotic protein agamous controls late stamen development by regulating a jasmonate biosynthetic gene in *Arabidopsis*. Plant Cell..

[45] Han P., García-Ponce B., Fonseca-Salazar G., Alvarez-Buylla E.R., Yu H. (2008). AGAMOUS-LIKE 17, a novel flowering promoter, acts in a FT-independent photoperiod pathway. Plant J..

[46] Mandel M.A., Yanofsky M.F. (1998). The *Arabidopsis* AGL9 MADS box gene is expressed in young flower primordia. Sex. Plant Reprod..

[47] Xu F., Dou J.S., Wang L.L., Yan J.P., Fu M.Y., Zhang X. (2017). Molecular Cloning and Expression Analysis of a AGAMOUS-like 66 Gene (GbAGL66) in Ginkgo biloba. Biotechnology.

[48] Mandaokar A., Thines B., Shin B., Lange B.M., Choi G., Koo Y.J., Yoo Y.J., Choi Y.D., Choi G., Browse J. (2006). Transcriptional regulators of stamen development in *Arabidopsis* identified by transcriptional profiling. Plant J..

[49] Shen X.P., Hu Z.W., Xiang X., Xu L.A., Cao J.S. (2019). Overexpression of a stamen-specific R2R3-MYB gene BcMF28 causes aberrant stamen development in transgenic *Arabidopsis*. Biochem. Biophys. Res. Commun..

[50] Millar A.A., Gubler F. (2005). The *Arabidopsis* GAMYB-like genes, MYB33 and MYB65, are microRNA -regulated genes that redundantly facilitate anther development. Plant Cell..

[51] Phan H.A., Iacuone S., Li S.F., Parish R.W. (2011). The MYB80 transcription factor is required for pollen development and the regulation of tapetal programmed cell death in *Arabidopsis* thaliana. Plant Cell..

[52] Yang C.Y., Song J., Ferguson A.C., Klisch D., Simpson K., Mo R., Taylor B., Mitsuda N., Wilson Z.A. (2017). Transcription factor MYB26 is key to spatial specificity in anther secondary thickening formation. Plant Physiol..

[53] Ghelli R., Brunetti P., Napoli N., De P.A., Cecchetti V., Tsuge T., Serino G., Matsui M., Mele G., Rinaldi G. (2018). A newly identified flower-specific splice variant of AUXIN RESPONSE FACTOR8 regulates stamen elongation and endothecium lignification in *Arabidopsis*. Plant Cell..

[54] Zhang Z.B., Zhu J., Gao J.F., Wang C., Li H., Li H., Zhang H.Q., Zhang S., Wang D.M., Wang Q.X. (2007). Transcription factor AtMYB103 is required for another development by regulating tapetum development, callose dissolution and exine formation in *Arabidopsis*. Plant J..

[55] Heisler M.G., Atkinson A., Bylstra Y.H., Walsh R., Smyth D.R. (2001). SPATULA, a gene that controls development of carpel margin tissues in *Arabidopsis*, encodes a bHLH protein. Development.

[56] Michael G., Teodora P., David R.S. (2008). Functional domains of SPATU-LA, a bHLH transcription factor involved in carpel and fruit development in *Arabidopsis*. Plant J..

[57] Gremski K., Ditta G., Yanofsky M.F. (2007). The HECATE genes regulate female reproductive tract development in *Arabidopsis thaliana*. Development.

[58] Zhu J., Lou Y., Xu X.F., Yang Z.N. (2011). A genetic pathway for tapetum development and function in *Arabidopsis*. J. Integr. Plant Biol..

[59] Gu J.N., Zhu J., Yu Y., Teng X.D., Lou Y., Xu X.F., Liu J.L., Yang Z.N. (2014). DYT1 directly regulates the expression of TDF1 for tapetum development and pollen wall formation in *Arabidopsis*. Plant J..

[60] Li W., Wang H.P., Yu D.Q. (2016). *Arabidopsis* WRKY transcription factors WRKY12 and WRKY13 oppositely regulate flowering under short-day conditions. Mol. Plant..

[61] Yu Y.C., Hu R.B., Wang H.M., Cao Y.P., He G., Fu C.X., Zhou G.K. (2013). MlWRKY12, a novel Miscanthus transcription factor, participates in pith secondary cell wall formation and promotes flowering. Plant Sci..

[62] Yu Y.C., Liu Z.H., Wang L., Kim Sang G.Y., Seo P.J., Qiao M., Wang N., Li S., Cao X.F., Park C.-M. (2016). WRKY 71 accelerates flowering via the direct activation of FLOWERING LOCUS T and LEAFY in *Arabidopsis thaliana*. Plant J..

[63] Zhang L.P., Chen L.G., Yu D.Q. (2018). Transcription factor WRKY75 interacts with DELLA proteins to affect flowering. Plant Physiol..

[64] Ma Z.B., Li W., Wang H.P., Yu D.Q. (2020). WRKY transcription factors WRKY12 and WRKY13 interact with SPL10 to modulate age-mediated flowering. Integr. Plant Biol..

[65] Yang L.L., Qi S.L., Touqeer A., Li H.Y., Zhang X.L., Liu X.F., Wu S. (2020). SlGT11 controls floral organ patterning and floral determinacy in tomato. BMC Plant Biol..

[66] Iván F.A., Hélène L., Sandra P.R., Eric S., Mats H., Mottinger J.P., Moreno M.A., Dellaporta S.L. (2009). Tasselseed1 is a Lipoxygenase Affecting Jasmonic Acid Signaling in Sex Determination of Maize. Science.

[67] Yadav S.R., Khanday I., Majhi B.B., Veluthambi K., Usha V. (2011). Auxin-responsive OsMGH3, a common downstream target of OsMADS1 and OsMADS6, controls rice floret fertility. Plant Cell Physiol..

[68] Huang H., Gao H., Liu B., Qi T.C., Tong J.H., Xiao L.T., Xie D.X., Song S.S. (2017). *Arabidopsis* MYB24 regulates jasmonate-mediated stamen development. Front. Plant Sci..

[69] Niwa T., Suzuki T., Takebayashi Y., Ishiguro R., Higashiyama T., Sakakibara H., Ishiguro S. (2018). Jasmonic acid facilitates flower opening and floral organ development through the upregulated expression of SlMYB21 transcription factor in tomato. Biosci. Biotechnol. Biochem..

[70] Mandaokar A., Browse J. (2009). MYB108 acts together with MYB24 to regulate jasmonate-mediated stamen maturation in *Arabidopsis*. Plant Physiol..

[71] Sun B.M., Zhu Z.S., Chen C.G., Chen G.J., Cao B.H., Chen C.M., Lei J.J. (2019). Jasmonate inducible R2R3-MYB transcription factor regulates capsaicinoid biosynthesis and stamen development in *Capsicum*. J. Agric. Food Chem..

[72] Wang B., Xue J.S., Yu Y.H., Liu S.Q., Zhang J.X., Yao X.Z., Liu Z.X., Xu X.F., Yang Z.N. (2017). Fine regulation of ARF17 for another development and pollen formation. BMC Plant Biol..

[73] Xu X.F., Wang B., Feng Y.F., Xue J.S., Qian X.X., Liu S.Q., Zhou J., Yu Y.H., Yang N.Y., Xu P. (2019). AUXIN RESPONSE FACTOR17 directly regulates MYB108 for anther dehiscence. Plant Physiol..

[74] Coleman H.D., Beamish L., Reid A., Park J.-Y., Mansfield S.D. (2010). Altered sucrose metabolism impacts plant biomass production and flower development. Transgenic Res..

[75] Jin S., Kim S.Y., Ahn J.H. (2017). Twin sister of FT (TSF) interacts with FRUCTOKINASE6 and inhibits its kinase activity in *Arabidopsis*. Front. Plant Sci..

[76] Wahl V., Ponnu J., Schlereth A., Arrivault S., Langenecker T., Franke A., Feil R., John E.L., Mark S., Markus S. (2013). Regulation of Flowering by Trehalose-6-Phosphate Signaling in Arabidopsis thaliana. Science.

[77] Xu F.Q., Li X.R., Ruan Y.L. (2008). RNAi-mediated suppression of nexokinasegene OsHXK10 in rice leads to non dehiscent anther and reduction of pollen germination. Plant Sci..

[78] David-Schwartz R., Liron W., Roee V., Hanita Z., Leonid M., Dvora S., David G. (2013). The SlFRK4 promoter is active only during late stages of pollen and anther development. Plant Sci..

[79] Sivitz A.B., Reinders A., Ward J.M. (2008). *Arabidopsis* sucrose transporter AtSUC1 is important for pollen germination and sucrose-induced anthocyanin accumulation. Plant Physiol..

[80] Goetz M., Guivarćh A., Hirsche J., Bauerfeind M.A., González M.C., Hyun T.K., Eom S.H., Chriqui D., Engelke T., Großkinsky D.K. (2017). Metabolic control of tobacco pollination by sugars and invertases. Plant Physiol..

